# Phosphorylated alpha‐synuclein in Iba1‐positive macrophages in the skin of patients with Parkinson's disease

**DOI:** 10.1002/acn3.51610

**Published:** 2022-06-24

**Authors:** Hideki Oizumi, Kenshi Yamasaki, Hiroyoshi Suzuki, Saki Ohshiro, Yuko Saito, Shigeo Murayama, Yoko Sugimura, Takafumi Hasegawa, Kohji Fukunaga, Atsushi Takeda

**Affiliations:** ^1^ Department of Neurology National Hospital Organization Sendai Nishitaga Hospital Sendai Japan; ^2^ Department of Dermatology Tohoku University Graduate School of Medicine Sendai Japan; ^3^ Department of Pathology and Laboratory Medicine National Hospital Organization Sendai Medical Center Sendai Japan; ^4^ Department of Pathology Tokyo Metropolitan Geriatric Hospital Tokyo Japan; ^5^ Department of Neurology Tohoku University Graduate School of Medicine Sendai Japan; ^6^ Department of Pharmacology Tohoku University Graduate School of Pharmaceutical Sciences Sendai Japan; ^7^ Department of Cognitive and Motor Aging Tohoku University Graduate School of Medicine Sendai Japan

## Abstract

**Background:**

Increasing evidence suggests that alpha‐synuclein (αSyn) accumulation in cholinergic and adrenergic fibers in the skin is a useful biomarker to diagnose idiopathic Parkinson's disease (IPD). It has been widely reported that phosphorylated αSyn (p‐αSyn) deposits in autonomic fibers in IPD are a biomarker in the skin, but other tissue localizations have not been fully investigated.

**Objective:**

It has been previously suggested that αSyn aggregates activate peripheral macrophages and that peripheral macrophages ingest pathological αsyn aggregates in aged rats or IPD patients. However, it remains to be elucidated whether peripheral macrophages in the skin of IPD patients accumulate αSyn. We evaluated whether (1) p‐αSyn deposits in dermal macrophages might represent a useful biomarker for IPD and (2) dermal macrophages play a role in the underlying pathogenesis of IPD.

**Methods:**

We performed an immunohistological analysis of skin biopsy specimens from IPD patients and controls.

**Results:**

We found that (1) p‐αSyn accumulation is present in dermal macrophages in skin biopsy specimens from patients with IPD, (2) not only dermal adrenergic fibers with p‐αSyn deposits but also dermal macrophages with p‐αSyn deposits are useful biomarkers for IPD patients and (3) the number of macrophages was significantly positively correlated with the number of macrophages with p‐αSyn deposits in the dermis of IPD patients.

**Interpretation:**

Our results suggest that dermal macrophages, which are innate immune cells, play an important role in IPD patients and are a novel biomarker for IPD.

## Introduction

Idiopathic Parkinson's disease (IPD) is a common secondary neurodegenerative disorder that affects more than 1% of the population over 65 years of age.^1^ The routine diagnosis of IPD depends on clinical criteria mainly focused on motor symptoms.^2^ However, the clinical criteria are not sufficient to precisely differentiate IPD from other parkinsonisms.^2^, ^3^

The histopathological features of Parkinson's disease (PD) are the loss of dopaminergic (DA) neurons in the substantia nigra (SN) and the presence of cytoplasmic protein aggregates known as Lewy bodies (LBs).^2^ The major components of LBs are β‐sheet fibrillar aggregates of alpha‐synuclein (αSyn) associated with synaptic vesicles in presynaptic nerve terminals.^4^ Intracellular αSyn forms aggregates within neurons but also spreads out into extracellular spaces.^5^, ^6^ Notably, Braak, Del Tredici^7^ postulated that a neurotoxic pathogen, possibly a viral toxin, may enter the brain via two routes: (1) A nasal route, with anterograde progression into the limbic system, and (2) a gastric route, after swallowing nasal secretions in saliva.^7^, ^8^, ^9^ The latter nasal secretions might contain a neurotoxic pathogen that, after penetrating the epithelial lining, could enter the axons of Meissner's plexus and reach the preganglionic parasympathetic motor neurons of the vagus nerve via transsynaptic transmission. These processes are hypothesized to be followed by retrograde transport into the medulla, pons, and midbrain, and finally, the SN might be affected.^7^, ^8^, ^9^ Thus, stereotypical cell‐to‐cell spreading of αSyn is believed to contribute to disease progression.

Several lines of evidence show that microglia are associated with the removal of extracellular αSyn aggregates and the response to neurodegeneration in the early stages of IPD pathogenesis.^5^, ^6^, ^10^, ^11^, ^12^ Although a correlation between αSyn aggregates and microglia is speculated in IPD pathogenesis, the phagocytosis of αSyn aggregates by microglia has not been demonstrated in human brain tissues. In this study, we examined possible relationships among neurodegeneration, microglia, and αSyn aggregates in brain autopsy specimens from IPD patients.

The dermal macrophages in the skin as well as the microglia in the brain are phagocytic cells. Dermal macrophages are involved in peripheral inflammation in innate immunity, autoinflammation, and acquired immunity and are involved in cutaneous tissue remodeling.^13^ Furthermore, it has been suggested that αSyn aggregates activate peripheral macrophages and that peripheral macrophages ingest pathological αsyn aggregates in aged rats or PD patients.^14^, ^15^ Although phosphorylated αSyn (p‐αSyn) deposits have been observed in sympathetic adrenergic and cholinergic fibers in the skin of IPD patients^16^, ^17^, ^18^ and cholinergic fibers have been observed in the rectal submucosa of IPD patients,^19^ whether p‐αSyn deposits are observed in macrophages in the skin has not been previously reported. In this report, we demonstrated that dermal macrophages in skin biopsy specimens from IPD patients contain p‐αSyn deposits.

## Materials and Methods

### Clinical information of the patients for skin biopsy

We studied 10 patients with IPD who met the clinical diagnostic criteria of the UK PD Society Brain Bank.^2^ The ages of the patients ranged from 56 to 77 years, with an average of 67.8 years. The 10 patients included four female and six male patients (Table 1). All patients had late‐onset disorders (older than 45 years), and none of them had a family history of PD. All patients were treated with L‐dopa alone or with other antiparkinsonian medications and maintained good control (CN) of their motor symptoms. We examined four consecutive autopsied decedents located at the Tokyo Metropolitan Geriatric Hospital as healthy CNs. The ages of the CNs ranged from 65 to 70 years, with an average of 67.8 years. The four CNs included two female and two male decedents (Table 1).

**Table 1:** Demographic profiles of IPD patients and CNs from whom skin biopsy specimens were collected.

Case	Participant	Sex	Age	Duration	MMSE
1	PD	Female	56	8	29
2	PD	Male	77	3	27
3	PD	Male	70	4	26
4	PD	Female	67	2	27
5	PD	Female	74	20	27
6	PD	Male	72	3	23
7	PD	Male	62	10	30
8	PD	Male	65	8	29
9	PD	Female	74	8	28
10	PD	Male	61	2	29
11	CN	Male	66		NE
12	CN	Male	70		NE
13	CN	Female	70		NE
14	CN	Female	65		NE

IPD, idiopathic Parkinson's disease; CN, control; PD, Parkinson's disease; NE, not examined; MMSE, mini mental state examination score.

#### Brain tissue from autopsied decedents

We examined eight consecutive autopsied decedents located at the National Hospital Organization Sendai Medical Center. Four of the decedents were diagnosed with IPD, and four of the decedents were used as CNs (Table 2). CNs were defined as follows: clinical symptoms were not observed, and abnormal p‐αSyn, amyloid β or phosphorylated tau aggregates were not observed in brain specimens from autopsied individuals. The ages of the decedents ranged from 62 to 87 years, with an average of 74.8 years. The entire brain tissue was fixed in 20% buffered formalin (Wako, Osaka, Japan) for 5–11 days. Serial coronal sections were dehydrated in a graded ethanol series, cleared in xylene, and embedded in paraffin using an automated tissue processor.

**Table 2:** Demographic profiles of patients from whom autopsy samples were collected.

Case	Participant	Age	Sex	Duration	CNS LB stage
1	PD	75	Male	Unknown	IV
2	PD	68	Female	10	IV
3	PD	87	Female	9	V
4	PD	75	Female	17	V
5	CN	62	Male		
6	CN	79	Male		
7	CN	83	Female		
8	CN	69	Male		

PD, Parkinson's disease; CN, control; CNS, central nervous system; LB, Lewy body.

#### Biopsied skin tissue

According to a previous report suggesting that postmortem skin specimens from patients with Braak stage III PD demonstrated the highest incidence of dermal p‐αSyn deposits,^16^ we recruited cognitively normal to mildly impaired IPD patients in the relatively early stage of IPD (Table 1). Skin specimens were collected from the cervical C8 paravertebral area, that is, close to the spinal ganglia,^17^ and from an axilla in IPD patients. Skin specimens were collected from an upper arm in CNs. Skin biopsies were performed using a 3‐mm disposable punch biopsy kit under sterile conditions after topical anesthesia with lidocaine. In IPD and CNs, skin biopsy specimens were fixed in 4% paraformaldehyde for 24–48 h.

### Histological immunofluorescence (IF) staining

Serial coronal 5‐μm slices were dehydrated in a graded ethanol series, cleared in xylene, and embedded in paraffin using an automated tissue processor. Mouse anti‐phosphorylated Ser129 alpha‐synuclein (p‐αSyn) monoclonal antibody (Immunostar, Huston, WI) was used to detect αSyn deposits in the skin or αSyn aggregates in the brain. To increase the sensitivity of detection of LB‐related p‐αSyn aggregates in the brain, brain sections were pretreated with formic acid. Rabbit anti‐dopamine beta hydroxylase (DBH) polyclonal antibodies (Abcam, ab209487, Cambridge, MA) were used to detect adrenergic fibers in the skin. To detect microglia in the brain, rabbit anti‐Iba1 polyclonal antibodies (Wako Chemicals, Richmond, VA) were used. To detect macrophages in the skin, rabbit anti‐Iba1 polyclonal antibodies (Wako Chemicals, Richmond, VA) and rabbit anti‐CD163 polyclonal antibodies (Abcam, ab189915, Cambridge, MA) were used. To increase the sensitivity of detection of CD163‐ir positive macrophages, skin sections were pretreated with target retrieval solution pH 9 (Agilent Technologies, Santa Clara, CA). To detect cytoplasmic autophagy‐associated proteins, rabbit anti‐p62/SQSTM1 polyclonal antibodies (1:100, ProteinTech, Rosemont, IL) were used.^20^ The sections were double‐immunostained overnight with combinations of primary antibodies: (1) Mouse anti‐p‐αSyn (1:1000 dilution) and rabbit anti‐DBH (1:100 dilution), (2) mouse anti‐p‐αSyn (1:1000 dilution) and rabbit anti‐Iba1 (1:100 dilution), (3) mouse anti‐p‐αSyn (1:1000 dilution) and rabbit anti‐CD163 (1:100 dilution) or (4) mouse anti‐p‐αSyn (1:1000 dilution) and rabbit anti‐p62/SQSTM1 (1:100 dilution). The sections were then washed and incubated with secondary antibodies for 2 h. The secondary antibodies anti‐mouse Alexa Fluor 488, anti‐rabbit Alexa Fluor 594, anti‐mouse Alexa Fluor 594, and anti‐rabbit Alexa Fluor 488 were obtained from Jackson ImmunoResearch (West Grove, PA) and were used at a 1:500 dilution. Then, the sections were treated with a TrueBlack™ lipofuscin autofluorescence quencher (Biotium, Hayward, CA) since brain tissue contains abundant lipofuscin. The sections were initially viewed and analyzed under a fluorescence microscope system (BZ‐X700, Keyence, Osaka, Japan).

### Quantitative analysis of immunostaining

#### Quantitative analysis of IF staining

To examine the number of macrophages and macrophages containing p‐αSyn deposits in skin sections, we measured the number of p‐αSyn‐immunoreactive (ir) positive deposits, Iba1‐ir positive macrophages, and Iba1‐ir and p‐αSyn double‐positive macrophages under 20‐fold magnified fields in the dermis in the region of interest of one section. To examine the numbers of adrenergic fibers and adrenergic fibers containing p‐αSyn deposits in skin sections, we measured the numbers of p‐αSyn‐ir positive deposits, DBH‐ir adrenergic fibers, and DBH‐ir and p‐αSyn double‐positive adrenergic fibers under 40‐fold magnified fields in the dermis in the region of interest of one section. To examine microglial cells, p‐αSyn aggregates and microglial cells containing p‐αSyn aggregates in brain autopsy specimens, we identified Iba1‐ir positive microglial cells, αSyn‐ir positive aggregates, and p‐αSyn‐ir and Iba1‐ir double‐positive microglial cells under 20‐fold magnified fields in the region of interest. For the analysis of the entorhinal cortex (EC) and amygdala, the gray matter area in the same region was defined as the region of interest.

#### Quantitative analysis of neuronal loss with hematoxylin and eosin staining

To evaluate neuronal loss in brain autopsy specimens, we counted the number of nuclei in neurons by hematoxylin and eosin (H&E) staining under 20‐fold magnified fields. The nuclei of neurons were identified morphologically by hematoxylin staining.

### Statistical evaluation

All values are expressed as the median (interquartile range). Significant differences between groups were identified using Wilcoxon rank sum test. Spearman's rank correlation coefficient was used to correlate the number of macrophages and the number macrophages with of p‐αSyn deposits. The data were analyzed with a JMP13 computer software system (SAS Institute, Tokyo, Japan). A *p* < 0.05 denoted a statistically significant difference.

## Results

### Microglia with p‐αSyn aggregates in brain autopsy specimens

**Figure 1:**
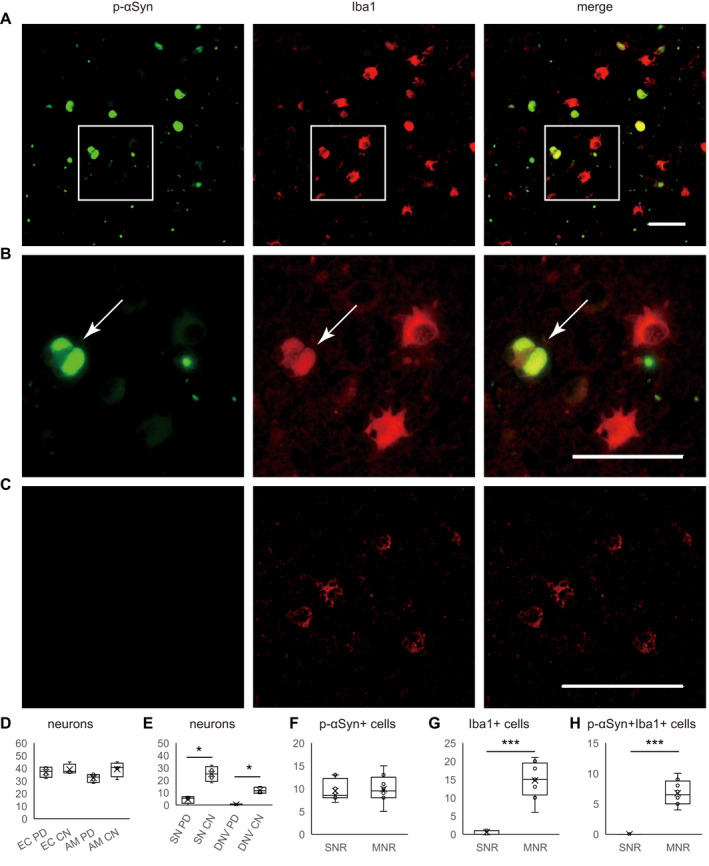
p‐αSyn aggregate‐positive microglia and neurons in brain autopsy specimens from PD patients. IF staining of Iba‐ir microglia (red) and p‐αSyn‐ir aggregates (green) in the EC classified as the mild neuronal loss region of IPD patients and CNs. Low (A) and high (B) magnification images are shown in the EC of IPD patients. High (C) magnification images are shown in the EC of CNs. Quantitative analysis of the numbers of neurons in the EC and AM of four IPD patients and four CNs (D). Quantitative analysis of the numbers of neurons in the SN and DNV of four IPD patients and 4 CNs (E). Quantitative analysis of the numbers of p‐αSyn aggregate‐positive cells (F), Quantitative analysis of the numbers of Iba1‐positive activated microglial cells (G), and Iba1‐positive microglial cells with p‐αSyn aggregates in the SNR and MNR of four IPD patients (H). IF images show Iba1‐positive activated microglia with p‐αSyn aggregates (A and B) in the EC of IPD patients but not CNs (C). In IPD patients, EC (*p* = 0.665) and AM (*p* = 0.108) were classified as the MNR because there was no neuronal loss compared to CNs (D). In IPD patients, SN (*p* < 0.05) and DNV (*p* < 0.05) were classified as the SNR because of the reduced number of neurons compared to CNs (E). LB‐related p‐αSyn aggregate‐positive cells were observed in both the SNR and MNR of IPD patients (*p* = 0.668) (F). Iba1‐positive activated microglia (amoeboid form) were more frequently observed in the MNR compared to SNR of IPD patients (*p* < 0.001) (G). Moreover, Iba1‐positive microglial cells with p‐αSyn aggregates were observed in the MNR but not SNR of brain specimens from IPD patients (*p* < 0.001) (H). ***p < 0.001, *p < 0.05. Scale bars: 50 μm (A–C). Circles indicate the data points between the lower and upper whiskers, and x indicates the average marker in a box/whisker diagram. p‐αSyn, phosphorylated alpha‐synuclein; IF, immunofluorescence; EC, entorhinal cortex; IPD, idiopathic Parkinson's disease; CNs, controls; SN, substantia nigra; SNR, severe neuronal loss regions; MNR, mild neuronal loss regions; AM, amygdala; LB, Lewy body; DNV, dorsal motor nucleus of the vagus nerve. [Colour figure can be viewed at wileyonlinelibrary.com]

To identify microglia with LB‐related αSyn aggregates, a double‐labeling IF study was performed by incubating sections of brain autopsy specimens with anti‐p‐αSyn mouse monoclonal antibody and anti‐Iba1 rabbit polyclonal antibodies. Iba1 is a 17‐kDa EF‐hand protein that is specifically expressed in microglia and macrophages and upregulated during immune activation.^21^ In IPD patients, EC (the number of neurons, 4 IPD vs. 4 CNs, 37.5 [36.25–43.25] vs. 37.5 [32.5–40.75]; [*p* = 0.665]) and amygdala (AM) (the number of neurons, 4 PD vs. 4 CNs, 33 [28.75–35] vs. 40.5 [33.25–44]); [*p* = 0.108]) were classified as the mild neuronal loss regions (MNR) because there was no neuronal loss in IPD patients compared to CNs in these regions (Fig. 1D). In IPD patients, SN (the number of neurons, 4 IPD vs. 4 CNs, 5.5 [1.25–6.75] vs. 25 [19–31]; [*p* < 0.05]) and dorsal motor nucleus of the vagus nerve (the number of neurons, 4 IPD vs. 4 CNs, 0 [0–0.75] vs. 11.5 [9.5–14.25]; [*p* < 0.05]) were classified as the severe neuronal loss regions (SNR) because of the reduced number of neurons in IPD patients compared to CNs in these regions (Fig. 1E).

Iba1‐positive microglial cells with p‐αSyn aggregates in brain autopsy specimens from IPD patients and CNs were analyzed. Interestingly, Iba1‐positive microglia with p‐αSyn aggregates were observed (Fig. 1A and B) in the EC of brain autopsy specimens from IPD patients but not CNs (Fig. 1C).

Next, LB‐related Iba1‐positive microglial cells and p‐αSyn aggregates in brain autopsy specimens from four IPD patients were analyzed in the SNR and MNR. LB‐related p‐αSyn‐positive cells were 155 observed in both the SNR and MNR (the number of p‐αSyn‐positive cells, 8.5 [8–12.25] vs. 9.5 [8–12.5]; [*p* = 0.668]) of brain autopsy specimens from four IPD patients (Fig. 1F). Iba1‐positive activated microglial cells (amoeboid form) were more abundant in the MNR compared to SNR (the number of Iba1‐positive activated microglial cells, 15 [10.75–19.5] vs. 1 [0–1]; [*p* < 0.001]) of brain autopsy specimens from four IPD patients (Fig. 1G). In addition, Iba1‐positive microglial cells with p‐αSyn aggregates were observed in the MNR but not SNR (the number of Iba1‐positive microglial cells with p‐αSyn aggregates, 6.5 [5–8.75] vs. 0; [*p* < 0.001]) of brain autopsy specimens from four IPD patients (Fig. 1H). Thus, in the brains of patients with IPD, the results suggest that Iba1‐positive microglia containing LB‐related αSyn aggregates are mainly localized in brain regions where neuronal cell death is not prominent. Our observation is in accordance with a previous report suggesting that αSyn aggregate‐related synaptic dysfunction may trigger later neurodegeneration in IPD.^22^

### Dermal macrophages with p‐αSyn deposits are markedly increased in skin specimens from IPD compared with those from CNs

To investigate the distribution of abnormal αSyn deposits in skin specimens from IPD patients, a single‐label IF study was performed by incubating skin sections from IPD patients with anti‐p‐αSyn (phospo‐Ser129, mouse monoclonal). p‐αSyn deposits were observed in both the posterior cervical C8 paravertebral and axillary skin of all IPD patients.

**Figure 2:**
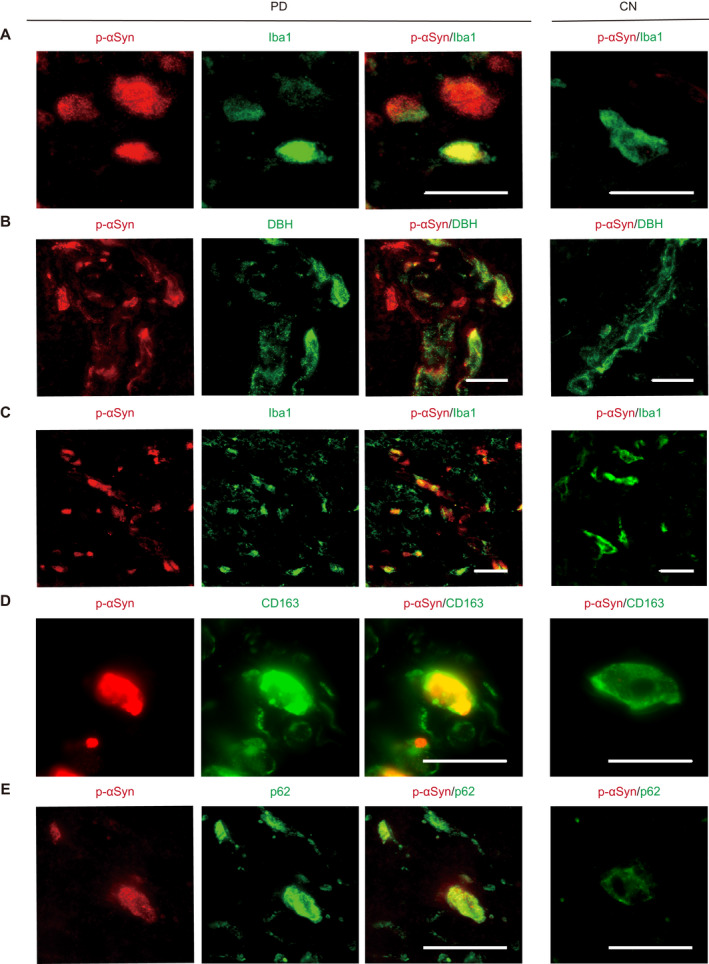
p‐αSyn deposits in macrophages and adrenergic sympathetic fibers in skin specimens from IPD patients. IF images of dermal p‐αSyn (red) and Iba1 (green)‐positive macrophages in the dermis of IPD patients and CNs (A and C). IF images of dermal p‐αSyn (red) and DBH‐ir positive vasomotor fibers (green) around blood vessels in the dermis of IPD patients and CNs (B). IF images of dermal p‐αSyn (red) and dermal CD163, another marker of macrophages, (green)‐positive macrophages in the dermis of IPD patients and CNs (D). IF images of dermal p‐αSyn (red) and p62/SQSTM1, cytoplasmic proteins associated with autophagy, (green)‐positive deposits in the dermis of IPD patients and CNs (E). IF images (high magnification) show Iba1‐ir positive macrophages with p‐αSyn deposits (A) and DBH‐ir adrenergic nerve fibers with p‐αSyn deposits around blood vessels (B) in the dermis of IPD patients but not CNs. IF images (low magnification) show that Iba1‐ir positive macrophages with p‐αSyn deposits were found diffusely in the dermis of IPD patients but not CNs (C). IF images (high magnification) show CD163‐ir positive macrophages with p‐αSyn deposits in the dermis of IPD patients but not CNs. (D). IF images (high magnification) show p‐αSyn‐ir positive deposits were colocalized with p62/SQSTM1 in the dermis of IPD patients but not CNs (E). Scale bars: 20 μm (A–E). IPD, idiopathic Parkinson's disease; IF, immunofluorescence; CNs, controls; DBH, dopamine beta hydroxylase; p‐αSyn, phosphorylated alpha‐synuclein. [Colour figure can be viewed at wileyonlinelibrary.com]

**Figure 3:**
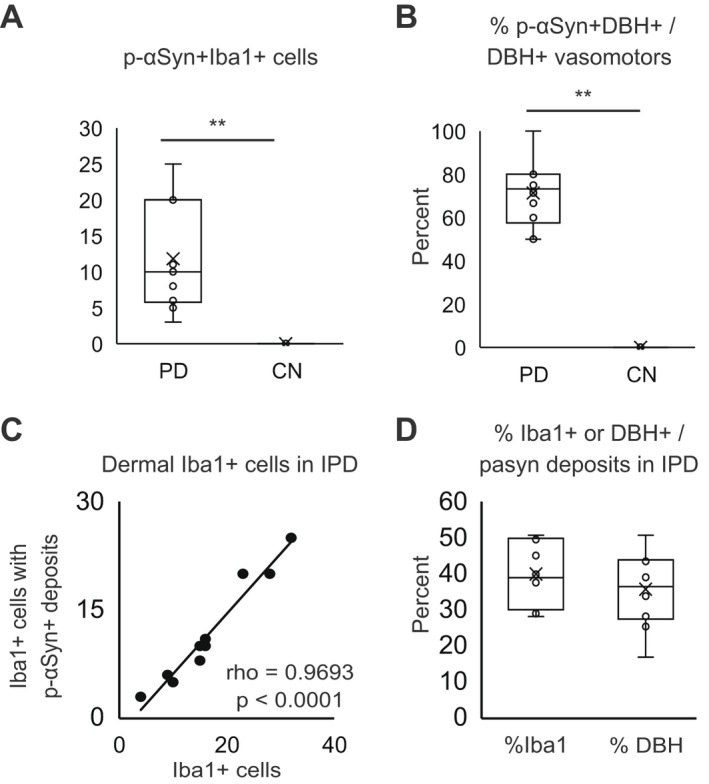
Quantitative analysis of dermal macrophages (mϕ) and adrenergic fibers with p‐αSyn deposits. Quantitative analysis of the number of p‐αSyn‐ir and Iba1‐ir double‐positive macrophages in the dermis of 10 IPD patients and four CNs (A). Quantitative analysis of the % p‐αSyn‐ir and DBH‐ir adrenergic fibers among DBH‐ir adrenergic fibers around arterioles in the dermis of 10 IPD patients and four CNs (B). Linear regression analysis was used to correlate the number of Iba1‐ir macrophages and the number of Iba1‐ir macrophages with p‐αSyn deposits in the dermis of 10 IPD patients (C). Quantitative analysis of the % p‐αSyn‐ir and Iba1‐ir double‐positive macrophages among p‐αSyn‐ir positive deposits and the % p‐αSyn‐ir and DBH‐ir double‐positive adrenergic fibers among p‐αSyn‐ir positive deposits in the dermis of 10 IPD patients (D). The number of p‐αSyn‐ir and Iba1‐ir double‐positive macrophages were observed in the dermis of IPD patients but not CNs (*p* < 0.01) (A). The % p‐αSyn‐ir and DBH‐ir adrenergic fibers among DBH‐ir adrenergic fibers were also elevated in the dermis of IPD patients but not CNs (*p* < 0.01) (B). The number of Iba1‐ir‐positive macrophages was significantly positively correlated with the number of Iba1‐ir macrophages with p‐αSyn deposits in the dermis of IPD patients (*p* < 0.0001) (C). There was no difference between the frequency of macrophages and adrenergic nerve fibers with p‐αSyn deposits in the dermis of IPD patients (*p* = 0.426) (D). Data from 10 IPDs and four CNs were analyzed. **p < 0.01. Circles indicate the data points between the lower and upper whiskers, and x indicates the average marker in a box/whisker diagram. IPD, idiopathic Parkinson's disease; CNs, controls; DBH, dopamine beta hydroxylase. p‐αSyn, phosphorylated alpha‐synuclein.

We identified Iba1‐ir microglial cells with p‐αSyn aggregates in the central nervous system (CNS) of PD patients. This suggests that microglial cells are directly involved in the degradation of αSyn aggregates. Thus, we hypothesized that the presence of p‐αSyn deposits in dermal Iba1‐positive macrophages, which are analogous to microglia, could be a biomarker based on immunological mechanisms in IPD. Based on this hypothesis, we examined whether macrophages in the axillary skin from IPD patients contained p‐αSyn deposits. The double‐labeling IF study with anti‐p‐αSyn mouse monoclonal antibody and anti‐Iba1 rabbit polyclonal antibodies revealed macrophages with p‐αSyn deposits in the dermis of IPD patients but not CNs (Fig. 2A). Iba1‐ir‐positive macrophages with p‐αSyn deposits were found diffusely in the dermis of IPD patients but not CNs (Fig. 2C). The double‐labeling IF study with anti‐p‐αSyn mouse monoclonal antibody and anti‐CD163, another marker of macrophages, rabbit polyclonal antibodies also revealed macrophages with p‐αSyn deposits in the dermis of IPD patients but not CNs (Fig. 2D). p‐αSyn deposits were colocalized with p62/SQSTM1, a cytoplasmic protein associated with autophagy, in the dermis of IPD patients but not CNs (Fig. 2E). The number of p‐αSyn‐ir and Iba1‐ir double‐positive macrophages in the dermis were observed in 10 IPD patients but not four CNs (the number of p‐αSyn‐ir and Iba1‐ir double‐positive cells, 10 [5.75–20] vs. 0; [*p* < 0.01]) (Fig. 3A). There was no difference in macrophages with p‐αSyn deposits staining pattern between the cervical C8 paravertebral and axillary skin in all IPD patients.

### Adrenergic nerve fibers with p‐αSyn deposits in skin specimens

In the skin, adrenergic DBH‐ir fibers mainly exist around arterioles. It is reported that p‐αSyn deposits in adrenergic fibers in the cervical C8 paravertebral skin from IPD patients.^23^ Thus, in this study double‐labeling IF studies were performed by incubating sections with anti‐p‐αSyn mouse monoclonal antibody and anti‐DBH rabbit polyclonal antibodies in the cervical C8 paravertebral skin sections obtained from IPD patients and CNs. The double‐labeling IF studies revealed the localization of p‐αSyn deposits in DBH‐ir adrenergic vasomotor fibers in the dermis of IPD patients but not CNs (Fig. 2B). p‐αSyn deposits in DBH‐ir adrenergic vasomotor fibers were mainly observed around blood vessels in the dermis of IPD patients. The % p‐αSyn‐ir and DBH‐ir double‐positive adrenergic fibers among DBH‐ir adrenergic fibers were observed in the dermis of 10 IPD patients but not four CNs (the % p‐αSyn‐ir and DBH‐ir double‐positive adrenergic fibers among DBH‐ir adrenergic fibers, 73.2 [57.5–80] vs. 0; [*p* < 0.01]) (Fig. 3B).

### Correlation between p‐αSyn deposits and macrophages in skin specimens from IPD patients

Spearman's rank correlation coefficient was used to correlate the number of Iba1‐ir macrophages and the number of Iba1‐ir macrophages with p‐αSyn deposits in the dermis of IPD patients. Interestingly, the number of Iba1‐ir macrophages was significantly positively correlated with the number of Iba1‐ir macrophages with p‐αSyn deposits in the dermis of IPD patients. (*ρ* = 0.9693, *p* < 0.0001) (Fig. 3C). These results suggest that the increase in αSyn deposition in dermal macrophages of IPD patients may be directly related to an increase in macrophages.

### Frequency of dermal macrophages and adrenergic nerve fibers with p‐αSyn deposits in skin specimens from IPD patients

We measured the % p‐αSyn‐ir and Iba1‐ir double‐positive macrophages among p‐αSyn‐ir positive deposits and the % p‐αSyn‐ir and DBH‐ir double‐positive adrenergic fibers among p‐αSyn‐ir positive deposits to compare the frequency of macrophages and adrenergic nerve fibers with p‐αSyn deposits in the dermis of IPD patients. There was no difference between the frequency of macrophages and adrenergic nerve fibers with p‐αSyn deposits in the dermis of 10 IPD patients (the % macrophages or adrenergic nerve fibers among p‐αSyn deposits, 38.4 [39.6–49.1] vs. 35.9 [27.1–43.3]; [*p* = 0.426]) (Fig. 3D). These results suggest that p‐Syn deposits were observed not only in adrenergic fibers but also in macrophages in the dermis.

## Discussion

### Microglia with p‐αSyn aggregates may indicate an early stage of neurodegeneration in IPD patients

The microglial cells containing p‐αSyn aggregates were mainly localized in brain regions where neuronal cell death was not remarkable. Activated microglia within the CNS are detectable using macrophage markers, including major histocompatibility complex II and Iba1.^13^, ^21^ Depending on the inflammatory environment, microglia exhibit both phenotypic and functional changes from resting to activating states. Activated microglia are amoeboid (larger cell bodies, shorter, and thicker processes) and are morphologically similar to the macrophages in peripheral tissues. A correlation between microglial activation and DA terminal loss in the affected nigrostriatal pathway in a PD mouse model also supports the hypothesis that neuroinflammatory responses induced by activated microglia contribute to the early neurodegeneration process.^24^, ^25^ Other previous reports also suggest that microglial activation is associated with the early stage of neurodegeneration in PD models.^5^, ^10^ In addition, microglia are capable of clearing extracellular αSyn aggregates by internalization and degradation in PD models.^5^, ^10^ Although microglial activation in PD models has been suggested, the presence of αSyn aggregates in the microglia of human PD brains has not been reported. In this study, p‐αSyn‐positive round structures within Iba1‐positive cells were observed in the MNR of PD patients but not CNs. Thus, for the first time, we demonstrated that LB‐related αSyn aggregates were observed in Iba1‐ir microglial cells of autopsy brain specimens from IPD patients. In addition, p‐αSyn‐positive round structures within Iba1‐negative cells were more observed in the SNR compared to MNR of IPD patients. On the other hand, p‐αSyn‐positive round structures within Iba1‐positive microglial cells were observed in the MNR but not SNR of IPD patients. Thus, microglia with αSyn aggregates may indicate an early stage of neurodegeneration in IPD patients. We also examined whether amyloid β or phosphorylated tau aggregates were observed in the MNR of IPD patients by using immunohistochemical analysis. We could not observe abnormal amyloid β or phosphorylated tau aggregates in the MNR of PD patients (data not shown). Thus, in this study, we suggested that there was no influence of concomitant amyloid β and tau pathologies on microglial activation in the MNR. In summary, our results suggest that the phagocytosis of p‐αSyn aggregates by microglia is a good marker for early‐stage synucleinopathies.

### p‐αSyn deposits as a potential useful biomarker for IPD

In this study, Iba1‐ir microglial cells with p‐αSyn aggregates in the CNS of IPD patients suggests that microglia are directly involved in the degradation of αSyn aggregates. Thus, we hypothesized that the presence of p‐αSyn deposits in dermal Iba1‐positive macrophages, which are analogous to microglia, could be a biomarker‐based on immunological mechanisms in IPD. Although αSyn accumulation in human induced pluripotent stem cell macrophages from PD patients has been observed,^15^ macrophages with p‐αSyn deposits in human skin tissues have not been reported. Therefore, we examined whether macrophages in the dermis of IPD contain p‐αSyn deposits. In this report, we first demonstrated dermal macrophages with p‐αSyn deposits in IPD. Moreover, we propose that not only dermal nerve terminals containing p‐αSyn deposits but also dermal macrophages with p‐αSyn deposits are good markers to differentiate IPD patients from CNs.

We also demonstrated that the number of Iba1‐ir macrophages significantly correlated with the number of Iba1‐ir macrophages with p‐αSyn deposits in the dermis in IPD patients. Increasing evidence suggests that the spread of αSyn is the underlying mechanism for LB‐related neurodegeneration.^5^, ^6^ In addition, it has been suggested that microglia in the CNS, which are analogous to macrophages, may clean extracellular αSyn and prevent the spread of αSyn‐related pathology.^26^ Phillips et al. showed that macrophages adjacent to dystrophic nerve terminals were swollen and contained insoluble αSyn in the myenteric plexus of aged rats.^14^ Haenseler et al. studied the removal role in αSyn using human induced pluripotent stem cell macrophages from PD patients. In this study, macrophages have the ability to remove fibrillary αSyn by phagocytosis, but high levels of exogenous or endogenous αSyn may reduce this ability and cause PD.^15^ These results suggest that macrophages play an active removal role in αSyn. Thus, our results indicated that Iba1‐ir peripheral macrophages were directly related to p‐αSyn deposits in the dermis in IPD patients in the present study.

In the present study, we demonstrated that p‐αSyn deposits in biopsied skin tissues of IPD patients were coexpressed with the cytoplasmic autophagy‐related protein p62. αSyn aggregates are also preferred targets for p62‐dependent autophagy in IPD.^27^, ^28^ Recently, αSyn aggregates with p62 accumulation were shown to be induced by autophagy dysfunction in DA neuron–specific autophagy‐deficient mice.^20^ Thus, our results suggested that p‐αSyn deposits with p62 accumulation in biopsied skin tissues of IPD patients might be degraded by the autophagy pathway.

Precise IPD diagnosis is important for predicting prognosis, selecting therapeutic options, and conducting appropriate clinical studies. To improve the accuracy of IPD diagnosis, several ongoing studies aim to identify biomarkers. Possible biomarkers include olfactory function, cerebral spinal fluid tests, and cardiac scintigraphy.^29^, ^30^, ^31^, ^32^, ^33^, ^34^ Since specific biomarkers have not yet been identified to distinguish IPD from other neurodegenerative parkinsonisms, a novel biomarker with high specificity and sensitivity is required. In the present study, dermal p‐αSyn deposits were observed in 10 (100%) of 10 IPD patients in the posterior cervical paravertebral skin. Several studies demonstrated that the incidence of dermal p‐αSyn staining in distal skin^18^, ^35^ was less than that in proximal skin in IPD patients.^17^, ^23^, ^36^, ^37^ Interestingly, dermal p‐αSyn deposits were observed in 80–100% of IPD patients when proximal cervical skin was used as the skin biopsy site.^17^, ^23^, ^36^, ^37^ Our results are in good accordance with previous studies demonstrating that cervical skin might be a promising biopsy site. We also observed dermal p‐αSyn deposits in the axillary skin of 10 (100%) of 10 IPD patients. Thus, we suggest that p‐αSyn deposits in axillary skin and posterior cervical skin might be a good target biomarker for IPD, and the former might be a more cosmetically suitable site for skin biopsy.

Here, we identified p‐αSyn deposits in the DBH‐ir adrenergic nerve vasomotor fibers of IPD patients. Our results were in accordance with several studies that observed dermal p‐αSyn deposits in postganglionic adrenergic fibers^16^, ^23^, ^35^ in IPD patients. Thus, the detection of p‐αSyn deposits in the skin autonomic fiber terminal may be a useful biomarker for IPD diagnosis.

### Limitations of this research

There are several limitations of this research. First, the number of samples was relatively small. Second, cross‐sectional longitudinal studies need to be performed. Third, and possibly the major limitation of this study, the IPD patients from whom skin biopsy specimens were taken were not genetically and pathologically diagnosed. Fourth, autopsy skin specimens of autopsy confirmed PD cases were not available, so IF study of these specimens could not be performed. Fifth, the skin biopsy samples are from different areas between IPD patients (C8 and axilla) and CNs (upper arm). We chose C8 and axilla to obtain highest incidence of dermal p‐αSyn deposits in IPD. Collaborators have reported that 149 of 1241 consecutive autopsy cases in the elderly were incidental LB.^38^ We considered that skin specimens with autopsy confirmed CNs were necessary for this study. Therefore, although skin biopsy samples are from different areas, the autopsy confirmed CNs obtained from the collaborator was included in this analysis. Sixth, this study was analyzed by IF staining only. Electron microscopic analysis was not performed. Thus, electron microscopy should be performed in future studies.

### Summary of the results

The main results of our study are as follows: (1) p‐αSyn deposits were detected in DBH‐ir adrenergic nerve vasomotor fibers and in Iba1‐positive macrophages in skin specimens; and (2) the number of macrophages was significantly positively correlated with the number of macrophages with p‐αSyn deposits in the dermis of IPD patients. These results suggest that p‐αSyn deposits in dermal macrophages, as well as adrenergic fibers are useful biomarkers to differentiate IPD patients from CNs and that peripheral macrophages are directly related to p‐αSyn deposits in the dermis in IPD. These results provide new insights into our understanding of pathophysiological processes in synucleinopathies.

## Conflict of Interest

The authors declare that the research was conducted in the absence of any commercial or financial relationships that could be construed as a potential conflict of interest.

## Author Contributions

Hideki Oizumi, Kenshi Yamasaki, Hiroyoshi Suzuki, Yuko Saito, Shigeo Murayama, Takafumi Hasegawa, Kohji Fukunaga and Atsushi Takeda designed the study; Hideki Oizumi, Kenshi Yamasaki, Hiroyoshi Suzuki, and Yuko Saito performed the experiments; Hideki Oizumi, Kenshi Yamasaki, and Hiroyoshi Suzuki analyzed the data; and Hideki Oizumi, Kenshi Yamasaki, Hiroyoshi Suzuki and Atsushi Takeda wrote the manuscript.

## References

[1] Ascherio A , Schwarzschild MA . The epidemiology of Parkinson's disease: risk factors and prevention. Lancet Neurol. 2016;15(12):1257‐1272. 27751556 10.1016/S1474-4422(16)30230-7

[2] Gibb WR , Lees AJ . The relevance of the Lewy body to the pathogenesis of idiopathic Parkinson's disease. J Neurol Neurosurg Psychiatry. 1988;51(6):745‐752. 2841426 10.1136/jnnp.51.6.745 PMC1033142

[3] Wirdefeldt K , Adami HO , Cole P , Trichopoulos D , Mandel J . Epidemiology and etiology of Parkinson's disease: a review of the evidence. Eur J Epidemiol. 2011;26(suppl 1):S1‐S58. 21626386 10.1007/s10654-011-9581-6

[4] Spillantini MG , Crowther RA , Jakes R , Hasegawa M , Goedert M . Alpha‐synuclein in filamentous inclusions of Lewy bodies from Parkinson's disease and dementia with Lewy bodies. Proc Natl Acad Sci USA. 1998;95(11):6469‐6473. 9600990 10.1073/pnas.95.11.6469 PMC27806

[5] Lee HJ , Suk JE , Bae EJ , Lee SJ . Clearance and deposition of extracellular alpha‐synuclein aggregates in microglia. Biochem Biophys Res Commun. 2008;372(3):423‐428. 18492487 10.1016/j.bbrc.2008.05.045

[6] Lee HJ , Bae EJ , Lee SJ . Extracellular alpha‐synuclein‐a novel and crucial factor in Lewy body diseases. Nat Rev Neurol. 2014;10(2):92‐98. 24468877 10.1038/nrneurol.2013.275

[7] Braak H , Del Tredici K , Bratzke H , Hamm‐Clement J , Sandmann‐Keil D , Rub U . Staging of the intracerebral inclusion body pathology associated with idiopathic Parkinson's disease (preclinical and clinical stages). J Neurol. 2002;249(suppl 3):III/1‐III/5. 10.1007/s00415-002-1301-4 12528692

[8] Hawkes CH , Del Tredici K , Braak H . Parkinson's disease: a dual‐hit hypothesis. Neuropathol Appl Neurobiol. 2007;33(6):599‐614. 17961138 10.1111/j.1365-2990.2007.00874.x PMC7194308

[9] Hawkes CH , Del Tredici K , Braak H . Parkinson's disease: the dual hit theory revisited. Ann N Y Acad Sci. 2009;1170:615‐622. 19686202 10.1111/j.1749-6632.2009.04365.x

[10] Su X , Federoff HJ , Maguire‐Zeiss KA . Mutant alpha‐synuclein overexpression mediates early proinflammatory activity. Neurotox Res. 2009;16(3):238‐254. 19526281 10.1007/s12640-009-9053-x PMC2877375

[11] Halliday GM , Stevens CH . Glia: initiators and progressors of pathology in Parkinson's disease. Mov Disord. 2011;26(1):6‐17. 21322014 10.1002/mds.23455

[12] Roodveldt C , Labrador‐Garrido A , Gonzalez‐Rey E , et al. Preconditioning of microglia by alpha‐synuclein strongly affects the response induced by toll‐like receptor (TLR) stimulation. PLoS One. 2013;8(11):e79160. 24236103 10.1371/journal.pone.0079160 PMC3827304

[13] Amici SA , Dong J , Guerau‐de‐Arellano M . Molecular mechanisms modulating the phenotype of macrophages and microglia. Front Immunol. 2017;8:1520. 29176977 10.3389/fimmu.2017.01520 PMC5686097

[14] Phillips RJ , Billingsley CN , Powley TL . Macrophages are unsuccessful in clearing aggregated alpha‐synuclein from the gastrointestinal tract of healthy aged Fischer 344 rats. Anat Rec (Hoboken). 2013;296(4):654‐669. 23441091 10.1002/ar.22675 PMC3851024

[15] Haenseler W , Zambon F , Lee H , et al. Excess alpha‐synuclein compromises phagocytosis in iPSC‐derived macrophages. Sci Rep. 2017;7(1):9003. 28827786 10.1038/s41598-017-09362-3 PMC5567139

[16] Ikemura M , Saito Y , Sengoku R , et al. Lewy body pathology involves cutaneous nerves. J Neuropathol Exp Neurol. 2008;67(10):945‐953. 18800013 10.1097/NEN.0b013e318186de48

[17] Donadio V , Incensi A , Leta V , et al. Skin nerve alpha‐synuclein deposits: a biomarker for idiopathic Parkinson disease. Neurology. 2014;82(15):1362‐1369. 24634456 10.1212/WNL.0000000000000316

[18] Zange L , Noack C , Hahn K , Stenzel W , Lipp A . Phosphorylated alpha‐synuclein in skin nerve fibres differentiates Parkinson's disease from multiple system atrophy. Brain. 2015;138(Pt 8):2310‐2321. 26017579 10.1093/brain/awv138

[19] Barrenschee M , Zorenkov D , Bottner M , et al. Distinct pattern of enteric phospho‐alpha‐synuclein aggregates and gene expression profiles in patients with Parkinson's disease. Acta Neuropathol Commun. 2017;5(1):1. 28057070 10.1186/s40478-016-0408-2 PMC5217296

[20] Sato S , Uchihara T , Fukuda T , et al. Loss of autophagy in dopaminergic neurons causes Lewy pathology and motor dysfunction in aged mice. Sci Rep. 2018;8(1):2813. 29434298 10.1038/s41598-018-21325-w PMC5809579

[21] Imai Y , Ibata I , Ito D , Ohsawa K , Kohsaka S . A novel gene iba1 in the major histocompatibility complex class III region encoding an EF hand protein expressed in a monocytic lineage. Biochem Biophys Res Commun. 1996;224(3):855‐862. 8713135 10.1006/bbrc.1996.1112

[22] Schulz‐Schaeffer WJ . The synaptic pathology of alpha‐synuclein aggregation in dementia with Lewy bodies, Parkinson's disease and Parkinson's disease dementia. Acta Neuropathol. 2010;120(2):131‐143. 20563819 10.1007/s00401-010-0711-0 PMC2892607

[23] Donadio V , Incensi A , Piccinini C , et al. Skin nerve misfolded alpha‐synuclein in pure autonomic failure and Parkinson disease. Ann Neurol. 2016;79(2):306‐316. 26606657 10.1002/ana.24567

[24] Furuya T , Hayakawa H , Yamada M , et al. Caspase‐11 mediates inflammatory dopaminergic cell death in the 1‐methyl‐4‐phenyl‐1,2,3,6‐tetrahydropyridine mouse model of Parkinson's disease. J Neurosci. 2004;24(8):1865‐1872. 14985426 10.1523/JNEUROSCI.3309-03.2004 PMC6730410

[25] Oizumi H , Hayashita‐Kinoh H , Hayakawa H , et al. Alteration in the differentiation‐related molecular expression in the subventricular zone in a mouse model of Parkinson's disease. Neurosci Res. 2008;60(1):15‐21. 17963913 10.1016/j.neures.2007.09.004

[26] Braczynski AK , Schulz JB , Bach JP . Vaccination strategies in tauopathies and synucleinopathies. J Neurochem. 2017;143(5):467‐488. 28869766 10.1111/jnc.14207

[27] Watanabe Y , Tatebe H , Taguchi K , et al. p62/SQSTM1‐dependent autophagy of Lewy body‐like alpha‐synuclein inclusions. PLoS One. 2012;7(12):e52868. 23300799 10.1371/journal.pone.0052868 PMC3534125

[28] Kuusisto E , Parkkinen L , Alafuzoff I . Morphogenesis of Lewy bodies: dissimilar incorporation of alpha‐synuclein, ubiquitin, and p62. J Neuropathol Exp Neurol. 2003;62(12):1241‐1253. 14692700 10.1093/jnen/62.12.1241

[29] Baba T , Takeda A , Kikuchi A , et al. Association of olfactory dysfunction and brain. Metabolism in Parkinson's disease. Mov Disord. 2011;26(4):621‐628. 21284041 10.1002/mds.23602

[30] Suzuki M , Hashimoto M , Yoshioka M , Murakami M , Kawasaki K , Urashima M . The odor stick identification test for Japanese differentiates Parkinson's disease from multiple system atrophy and progressive supra nuclear palsy. BMC Neurol. 2011;22(11):157. 10.1186/1471-2377-11-157 PMC3297535 22192419

[31] Doty RL . Olfactory dysfunction in Parkinson disease. Nat Rev Neurol. 2012;8(6):329‐339. 22584158 10.1038/nrneurol.2012.80

[32] Orimo S , Suzuki M , Inaba A , Mizusawa H . 123I‐MIBG myocardial scintigraphy for differentiating Parkinson's disease from other neurodegenerative parkinsonism: a systematic review and meta‐analysis. Parkinsonism Relat Disord. 2012;18(5):494‐500. 22321865 10.1016/j.parkreldis.2012.01.009

[33] Fairfoul G , McGuire LI , Pal S , et al. Alpha‐synuclein RT‐QuIC in the CSF of patients with alpha‐synucleinopathies. Ann Clin Transl Neurol. 2016;3(10):812‐818. 27752516 10.1002/acn3.338 PMC5048391

[34] Kakuda K , Ikenaka K , Araki K , et al. Ultrasonication‐based rapid amplification of alpha‐synuclein aggregates in cerebrospinal fluid. Sci Rep. 2019;9(1):6001. 30979935 10.1038/s41598-019-42399-0 PMC6461702

[35] Doppler K , Ebert S , Uceyler N , et al. Cutaneous neuropathy in Parkinson's disease: a window into brain pathology. Acta Neuropathol. 2014;128(1):99‐109. 24788821 10.1007/s00401-014-1284-0 PMC4059960

[36] Doppler K , Jentschke HM , Schulmeyer L , et al. Dermal phospho‐alpha‐synuclein deposits confirm REM sleep behaviour disorder as prodromal Parkinson's disease. Acta Neuropathol. 2017;133(4):535‐545. 28180961 10.1007/s00401-017-1684-z PMC5348554

[37] Donadio V , Incensi A , Rizzo G , et al. Spine topographical distribution of skin alpha‐synuclein deposits in idiopathic Parkinson disease. J Neuropathol Exp Neurol. 2017;76(5):384‐389. 28402459 10.1093/jnen/nlx021

[38] Saito Y , Ruberu NN , Sawabe M , et al. Lewy body‐related alpha‐synucleinopathy in aging. J Neuropathol Exp Neurol. 2004;63(7):742‐749. 15290899 10.1093/jnen/63.7.742

